# Glial pharmacology in Asia & Beyond

**DOI:** 10.1002/prp2.881

**Published:** 2021-10-22

**Authors:** Schuichi Koizumi

**Affiliations:** ^1^ Department of Neuropharmacology Graduate School of Medicine University of Yamanashi Yamanashi Japan; ^2^ GLIA Center University of Yamanashi Yamanashi Japan

In this special issue entitled “Glial pharmacology in Asia & beyond,” we provide an up‐to‐date overview of several aspects of glial pharmacology. With recent advances in brain science, the importance of glia in brain function is attracting more and more attention. Although glial research is increasing worldwide, this trend is particularly strong in Asia. It is well known that glial cells are important in the regulation of brain physiology, but their function in pathological conditions is particularly of interest. Thus, many researchers have started to study glial dysfunction in brain diseases, including research on glial cells in the control of sensory information, such as intractable pain and itch with translational research leading to drug discovery expected. In addition, research on glial differentiation and transcriptional regulation of glial cell function has produced many noteworthy results. Furthermore, there have been important achievements in the ex vivo manipulation of glial cells. Glial cells are electrophysiologically non‐excitable, it is not appropriate to manipulate their functions by optogenetics as is done in neurons. Instead, glial cell manipulation by designer receptors exclusively activated by designer drugs (DREADDs) is a promising method that elucidates the function of glial cells. Successful examples are included in this Special Issue.

The Special Issue starts with an overview of sensory controls by glial cells. Here Shiratori and Tsuda describe the dysfunction of spinal microglia and astrocytes in altering the quality and quantity of primary afferents, resulting in abnormal neurotransmission.^1^ This follows on from their discovery that neuropathic pain is initiated in spinal microglia and that pharmacological treatment strategies are available for its treatment.^2^, ^3^ Spinal glial cells play a central role not only in chronic pain but also in chronic itch. With regard to chronic itch, astrocytes in the spinal cord have a critical role.^4^ Itch is normally transient and disappearing gradually with temporary scratching to the affected area. However, it sometimes become chronic. Such chronic itch is caused by activation of spinal astrocytes which receive inflammatory signals such as interleukin‐6 (IL‐6) or IL‐33 from skin and sensory neurons, and increase a transcription factor signal transducer and activator of transcription 3 (STAT3). They also increase lipocalin 2 (LCN2) in a STAT3‐dependent manner to sensitize spinal itch neurons.^5^ These findings are expected to contribute significantly not only to the understanding of astrocyte‐dependent mechanisms of chronic itch, but also to the development of a new therapeutic strategy targeting astrocytes.

Plants have long been used not only as foods but also as drugs since ancient times. In fact, a quarter of the currently‐consumed drugs originate from plants. East Asian countries, such as China, Japan and Korea have developed these plant medicine or herbal medicine in their own unique ways. Takanashi et al., have extended their research on glial cells and chronic pain, summarize the effects of KAMPO, a herbal medicine unique to Japan, on neuropathy caused by cancer chemotherapy.^6^ The anti‐cancer agents platinum derivatives and taxanes such as paclitaxel (PCX) often cause neuropathy known as chemotherapy‐induced peripheral neuropathy with high frequency. They show new findings that Goshajinkigan (GJG), a Japanese KAMPO medicine, inhibits PCX‐induced neuropathy by inhibiting the activation of astrocytes in the primary sensory cortex (S1 cortex). This group previously demonstrated that neuropathic pain is caused by re‐wiring of tactile and pain circuits of S1 cortex.^7^, ^8^ To date, there are no or only limited number of chemicals that target glial cells. Thus this report by Takanashi et al. is interesting because it shows that a traditional medicine, KAMPO, can be applied to control completely new target glial cells. Lee et al. further extend the potential of a traditional herbal medicine, summarizing how various plants act on glial cells and actually can be available for the treatment of chronic pain. As mentioned above, Korea also has a long tradition of herbal medicine for pain treatment including chemotherapy‐induced peripheral neuropathy.^9^, ^10^, ^11^ Kim's group is also a leader of glia and pain research.^7^, ^8^ In the current report, they chose 27 typical formulas of medical herbs, including GJG, and single herbs, and also 21 representative phytochemicals, and describe how effective these agents work on several pain diseases.^7^, ^12^ Although detailed mechanisms and effective ingredients of medical herbs need to be studied more, these findings suggest that plant or herbal medicine in East Asia would be a promising therapeutic agent for glial‐dependent pain, as well as for disorders in the CNS. Overall, the research on glial cells for pain and itch, and their unique treatment strategies for these, will provide a new direction for future glial research and pharmacology of glial cells.

The functions of microglia are diverse and especially more pronounced in the condition of several brain disorders. Therefore, it is a very important issue to control microglial functions properly. For this, nuclear receptors have critical roles because they can strongly change the microglial phenotypes. Katsuki summarizes recent findings of nuclear receptor‐mediated control of microglia especially focusing on NR1 and NR4.^13^, ^14^ Nuclear receptors are divided into 7 subclasses, thyroid hormone receptor‐like (NR1 subfamily), retinoid X receptor‐like, estrogen receptor‐like, nerve growth factor‐inducible B protein‐like (NR4 subfamily), germ cell nuclear factor‐like and miscellaneous ones. Among these, NR1 and Nr4 subfamilies are relevant because ligands for NR1 subfamily such as vitamin D strongly inhibits proinflammatory responses in microglia, and similar inhibitions are observed in other NR1 subfamily receptors such as peroxisome proliferator‐activated receptors (PPARs) and liver X receptors (LXR).^15^ The work also shows the importance of NR4 subfamily such as Nur77 and Nurr1 for controlling microglial functions. Again, nuclear receptors greatly and effectively control microglial functions, and thus, would be a promising target for various brain diseases such as Alzheimer's disease, Parkinson's disease, and ischemic stroke. These data strongly suggest that nuclear receptors should become more important for future glial pharmacology.

The detailed mechanisms of glial cell differentiation remains unclear. Similar to neurons, macroglia, that is, astrocytes and oligodendrocytes are differentiated from neural stem/precursor cells (NS/PCs). NSs/PCs increase their number by a repeated self‐renewal, and then differentiate into neurons, followed by differentiation into oligodendrocytes and astrocytes. However, the mechanism of this switching is not well understood. Following on from colleagues work,^16^ Takouda et al. show that SRY‐box transcription factor 8 (Sox8), a SoxE group transcription factor, can function as a switch of the trains of responses and promote astrocytes generation from NS/PCs.^17^ They also show that Sox8 is a direct target gene of nuclear factor IA (Nfia) that controls gliogenesis. Since impairment of astrogenesis during development is associated with a variety of neurological disorders such as epilepsy and autism,^18^ a more detailed understanding of the mechanisms of astrogenesis may help with strategies for the treatment of these disorders.

Methods to clarify cell functions by manipulating cells such as optogenetics and chemogenetics have made significant achievements in functional biomedical research. In the field of glial research, chemogenetics with genetically modified G‐protein–coupled receptors (GPCRs), so‐called designer drugs (DREADDs) have greatly developed our understanding of glial functions. Among GPCRs in glial cells, functions of Gq‐GPCRs have been investigated, but Gi‐GPCRs have received only limited attention. In this issue, Kim et al. evaluated the role of Gi‐GPCR using Gi‐DREADD in the hippocampal astrocytes, following on from colleagues work in this area.^19^ Kim found that activation of Gi‐DREADD by clozapine N‐oxide (CNO) inhibits neuroinflammation in the hippocampus with decreases in proinflammatory cytokines, glial activation, and improvements in cognitive function.^20^ This lead to the conclusion that Gi‐GPCRs in hippocampal astrocytes play a role in the inhibition of neuroinflammation. Such manipulation techniques should further advance the understanding of the role of glial cells.

In summary, this special issue summarizes the latest findings on glia from pharmacologists working in Asia. It aims to stimulate interest in this field among a wider readership of the journal and to foster further research and drug discovery focused on glial cell biology, pathophysiology, and therapeutic solutions.
